# YAP1/Piezo1 involve in the dynamic changes of lymphatic vessels in UVR-induced photoaging progress to squamous cell carcinoma

**DOI:** 10.1186/s12967-023-04458-z

**Published:** 2023-11-16

**Authors:** Yuling L. Yang, Chu Zhou, Qi Chen, Shuzhan Z. Shen, Jiandan D. Li, Xiuli L. Wang, Peiru R. Wang

**Affiliations:** Institute of Photomedicine, Shanghai Skin Disease Hospital, School of Medicine, Tongji University, Shanghai, China

**Keywords:** Skin cancer, Photoaging, Ultraviolet radiation, Lymphatic vessels, Immune microenvironment

## Abstract

**Background:**

UV-induced cutaneous squamous cell carcinoma (cSCC) is one of the most common skin cancers. The constant alterations of the lymphatic-centered immune microenvironment are essential in transforming from photoaging to cSCC. Studying the mechanism will be beneficial for new targets exploration to the early prediction of cSCC.

**Aims:**

To investigate the dynamic changes and mechanism of the lymphatic-centered immune microenvironment in transforming from photoaging to cSCC induced by ultraviolet irradiation (UVR).

**Methods:**

TIMER2.0 was used to analyze whether YAP1/VEGFC signaling pathway is involved in lymphangiogenesis in head and neck squamous cell carcinoma (HNSCC). Meanwhile, lymphatic-centered immune microenvironments alterations and the related cumulative survival time were also analyzed. With the accumulated UVR, skin photoaging developed and gradually progressed into actinic keratosis and cSCC on SKH-1 hairless mice. The skin lymphatic-centered immune microenvironment was evaluated at the 0th, 8th, 12th, 16-18th, and 20-24th week of UVR. Skin phenotype was assessed using optical coherence tomography (OCT) and skin image. H&E and Masson’s trichrome staining evaluated epidermis and dermis. The structure of lymphatic vessels (LVs), blood vessels, and different types of T cells were evaluated by immunohistochemistry staining. The expression of Piezo1 whose deletion in adult lymphatics led to substantial valve degeneration, VE-cadherin that maintained the permeability of LVs, and YAP1 were evaluated by immunohistochemistry staining as well. Besides, the drainage function of LVs was assessed by Evans Blue assay in vivo.

**Results:**

The lymphatic function and immune cell infiltration underwent adaptive changes under continuous UVR. TIMER2.0 analysis indicated that VEGFC genes high expressed in HNSCC. YAP1 gene expression was positive correlated with VEGFC in HNSCC. LV density increased in human cSCC. More LVs in HNSCC were beneficial to prolong the survival time. VEGFC gene overexpression was positive correlated to CD8^+^T cell infiltration. More CD8A^+^T cells and CD8B^+^T cell infiltration in HNSCC extended survival time. When YAP1 gene overexpression and high infiltration of endothelial cells took place simultaneously might prolong the survival time of HNSCC patients. And high infiltration of CD8^+^T cells prolonged the survival time as well. In animal studies, UVR-induced eight weeks (photoaging) and 16–18 weeks (precancerous) were two turning points. The density of LVs in UV-8w was the least. When photoaged skin developed into AK lesions (UV-16-18w), LV slightly exceeded healthy skin and proliferated sharply in cSCC (UV-20-24w). YAP1 expression was almost consistent with LV but rose after the photoaging stage. The drainage of cSCC mice induced by UVR was better than that of photoaged skin and worse than that of health skin. The dynamic alterations of LVs number, Piezo1 expression, and collagen might be reasons for it. The expression of Piezo1 was in the highest point after 8 weeks of UVR, then gradually descended to the platform. The total T cells increased slowly, but the infiltration of CD4^+^T cells increased, and CD8^+^T cells decreased after eight weeks of UVR. The CD8^+^T cells and CD4^+^T cells increased sharply in UV-16-18w and UV-20-24w groups.

**Conclusion:**

The lymphatic-centered immune microenvironment underwent adaptive changes under continuous UVR via regulating YAP1/VEGFC and Piezo1. During the formation of cSCC, there are two turning points, eight weeks (photoaging) and 16–18 weeks (precancerous). YAP1, Piezo1, LVs, and immune cells constantly changed with the skin state induced by UVR. According to these changes the process of cSCC can be identified in advance and intervene timely.

## Key points

- It investigated the dynamic changes of the lymphatic-centered immune microenvironment firstly in cSCC formation process induced by UVR.
- The lymphatic-centered immune microenvironment has adapted under continuous UVR via regulating YAP1/VEGFC and Piezo1.
- There are two turning points of lymphatic changes occurred during UV-induced cSCC formation. YAP1, Piezo1, LVs, and immune cells constantly changed with the skin state induced by UVR. According to these changes, the process of cSCC can be identified in advance and intervene timely.

## Introduction

Ultraviolet irradiation (UVR) is one of the major causative factors for skin photoaging and cutaneous squamous cell carcinoma (cSCC) [1, 2]. The probability of each AK lesion progressing to cSCC within one year is 0.60%, but the probability increases to 2.57% within four years. 60% of cSCC patients have a AK medical history, and the malignant transformation rate of multiple AK is much higher than that of single AK lesion, and increases by 10% with age [3]. The percentage of first-time diagnosed cSCC patients which may experience recurrent cSCC within 5 years is 30–50% [4]. Preventing UV-induced precancerous lesions from developing into cSCC would save medical resources and relieve social pressure. Long-term exposure to UVB induces inflammation (senescence-associated secretory phenotype and other inflammatory factors), oxidative stress (reactive oxygen species, ROS), and DNA mutation, which are involved in the initiation, promotion, and progression of cSCC [5]. UVB-induced inflammation is an important and dynamic event in all stages of cSCC formation [6, 7], understanding the immune microenvironment dynamic changes and alleviating the UVB-induced inflammation to prevent skin aging and progressing into cutaneous cancers. A strong correlation exists between the lymphatic vessel (LV) and immune cell infiltration in human metastatic cancer [8]. T lymphocytes (T cells) are mainly divided into two functionally subgroups, CD4^+^ T helper cells (Th) and the CD8^+^ cytotoxic T lymphocytes (CTL). CD4^+^T cells assist humoral and cellular immunity. CD8^+^T cells have the function of killing target cells [9]. The role of lymphangiogenic structure in the composition of the microenvironment of a tumor is dual and highly complicated. On the one hand, LVs promote tumor progression by actively contributing to metastatic dissemination [10]; On the other hand, the anti-tumor immune response can be initiated only with preserved vascular function to control immune cell trafficking, adhesion, and homing [11]. The current realization is that promoting lymphangiogenesis, coupled with methods to enhance T cell-mediated immunity, may be especially efficacious for treating cancer while not increasing the risk of metastasis [11]. There was no conclusion on how the LVs change, whether they maintain their function to control immune cells and be beneficial for treating cancer during the transition from healthy skin to photoaging, actinic keratosis (AK), and cSCC.

The lymphatic vascular network is a low-pressure, unidirectional flow system in every body organ. LVs are lined with lymphatic endothelial cells (LECs), which express the receptor tyrosine kinase vascular endothelial growth factor receptor-3 (VEGFR-3) and lymphatic vessel endothelial hyaluronan receptor 1 (LYVE1) [12, 13]. LYVE1 is a protein that can be used to label LVs. VEGFR-3 and its secreted ligand, vascular endothelial growth factor C (VEGF-C), are the significant drivers of developmental and pathological lymphangiogenesis. VEGFC acts as the upstream of Yes-associated protein 1 (YAP1) in lymphangiogenesis to promote the proliferation of LVs [14]. Studies showed that YAP, an effective driver of cell proliferation as a critical regulator of the Hippo signaling pathway, was up-regulated in various tumor tissues, such as colon and breast cancer [15, 16]. Despite YAP1 playing essential roles in the LVs and the context of cSCC development, its parts remain unknown. The development and function maintenance of the lymphatic valve are regulated by Piezo-type mechanosensitive ion channel component 1 (Piezo1), a mechanically activated ion channel. Its deletion in adult lymphatics cause substantial valve degeneration [17, 18]. In recent reports, an aberrant expression of Piezo1 has been correlated with poor prognosis in sarcoma and bladder cancers [19–22]. It has yet to learn how Pizeo1 changes during the progression of cSCC induced by UVR.

Long-term UVR induces histopathological alterations in the lymphatic-centered immune microenvironment. The dysfunction of the pumping system and the immune function decline further accelerates the process of cell senescence and tumorigenesis. BVs and LVs determine a delicate and continuous interaction between tumor cells and tumor-infiltrating lymphocytes so that a light but dynamic balance is established, by which an anti- or pro-tumourigenic contexture is defined in the process of ‘immunoediting’ [23], thereby dictating disease outcome [24, 25]. However, few studies have focused on the changes in LV, BV, and the lymphatic-centered immune microenvironment in cSCC formation. Besides, Piezo1 and YAP1 play vital roles in maintaining the drainage function and contribute to the proliferation of LV. Still, there is no conclusion regarding their dynamic changes during the formation of cSCC. Currently, the main treatment methods for cSCC include surgical and non-surgical treatments. Non-surgical treatment comprises fluorouracil and imiquimod cream, cryotherapy and electric drying curettage, photodynamic therapy, targeted therapy (cetuximab), etc. Regardless of the treatment manners, problems such as high treatment costs and high recurrence rates exist [4]. Meanwhile, with the deepening of social aging, the prevalence of cSCC is increasing yearly [26, 27]. Monitoring the dynamic formation process of cSCC, evaluating the dynamic changes of the immune microenvironment, and timely intervention are conducive to reducing the recurrence rate and relieving the disease burden. Thus, this study aims to investigate the histopathological alterations in the lymphatic-centered immune microenvironment of SKH1 mice and to discover the turning points during the tumor formation induced by UVR, which provide a specific clinical significance for the early diagnosis of cSCC and judgment of prognosis.

## Method

### Animals

Hairless SKH-1 mice (female, 12 weeks old, immunocompetent) were obtained from Shanghai Public Health Clinical. Young SKH-1 mice (three months old) were randomly divided into five groups (UV-0w: n = 4, UV-8w: n = 4, UV-12w: n = 3, UV-16-18w: n = 4, and UV-20-24w: n = 4). UV-0w group mice didn’t receive UVR. UV-8w group mice received two months of UVR, and features of photoaging could be observed on the dorsal skin of mice, including coarse wrinkles, erythema, and scales. UV-12w group mice received three months of UVR. UV-16-18w group mice received 16 to 18 weeks of UVR until the AK or precancerous skin lesions appeared. AK manifests as erythema, keratotic maculopapular, and plaques, often covered with adhesive scabs on the surface. In the UV-20-24w group, chronic UVR (20–24 weeks of UVR) made the dorsal skin develop into tumors. cSCC manifests as nodules with unclear boundaries, which can quickly evolve into warty or papillomatous shapes. It is prone to necrosis and bleeding [4]. Besides, another 16 mice (16 weeks old, immunocompetent) were divided into three groups (UV-0w, UV-8w, UV-20-24w group, implant SCC) to evaluate the drainage function of LV. Evans Blue assay was applied in the UV-0w group (n = 4, four months old), UV-8w group (n = 4, received eight weeks of UVR), UV-20-24w group (n = 5, received 20–24 weeks of UVR), and implant-SCC group (n = 3, four months old) (Fig. 2C, D). All experiments followed guidelines and were approved by the Shanghai Skin Disease Hospital Animal Ethics Committee.

### Study design

AK and cSCC were induced by solar-simulated UVR (Solar UV Simulator, SIGMA, Shanghai, China) five times weekly for 24 weeks. According to the Zhou et al. paper, the concrete steps adopted the protocol described in the scheme [28].

### Histopathological examination

Skin tissues were collected and subjected to H&E (Haemotoxylin and Eosin), Masson’s trichrome staining, and other examinations. The epidermal thickness was measured vertically from the epidermal-dermal junction to the spinous layer at 4 random spots in each mice skin, and the dermal thickness was measured vertically from the epidermal-dermal junction to the top of the cyst layer. The cyst layer is one of the characteristic structures in the skin of SKH-1 mice. It locates beneath the dermis and contains lipids. Image Pro Plus software (Media Cybernetics) measured epidermis, dermis, and collagen thickness.

### Immunohistochemistry

Immunohistochemistry (Formalin/PFA-fixed paraffin-embedded sections) analysis of mice skin tissues labeling with LYVE1 (1:200; Abcam ab219556), CD31 (1:100; abcam, ab222783), CD3 (1:200; Abcam ab16669), CD4 (1:100; abcam, ab183635), VE-CAD (1:100, affinity, AF6265), Piezo1 (1:100; affinity, DF12083), YAP1 (1:100; CST, 14074S), and CD8 (1:100; abcam, ab217344) overnight in 4 °C refrigerator. Besides, immunohistochemistry analysis of human skin tissues labeling with LYVE1 (1:200; Abcam ab219556), CD31 (1:100; abcam, ab28364). Heat-mediated antigen retrieval was performed at 95 °C for 20 min. Blocking was performed using 5% BSA for 30 min at room temperature. Anti-rabbit HRP secondary antibodies were used as the secondary antibody. In the immunohistochemistry results statistics of each mouse, multiple fields of view were randomly selected under a 100 × microscope for statistical analysis (basically 3 or 4 regions), such as CD31, a mouse randomly selected from 4 fields, and LYVE1 randomly selected from 3 fields. According to the tissue staining, two histopathologists recorded positive results in each area using Image-Pro Plus software (Media Cybernetics).

### OCT imaging

We used the spectral-domain OCT (SD-OCT) imaging system (Ganymede-II-HR, Thorlabs, Newton, New Jersey). The highest A-scan rate of the system was 36 kHz. The axial resolution of SD-OCT was previously quantified as 4.0 μm, whereas the lateral resolution was 3.8 μm. The central wavelength was 900 nm, and the scan head (Thorlabs, OCTP-900) equipped with a scan lens (Thorlabs, OCT-LK3) was employed for OCT imaging [28].

### Evans blue assay

The Evans Blue (EB) assay was performed according to the protocol described in the Wang et al. paper [12].

### Implant SCC cells

To establish an implanted cSCC mouse model, XL50 cells (mouse cSCC cells; 5 × 10^6^) were injected subcutaneously into the backs of mice. After 14 days, the tumor-bearing mice were used for the study when the tumor volume reached approximately 7 mm in diameter [29].

### Immune cells infiltration analysis

TIMER2.0 was used to analyze the relationship between the lymphatic-centered immune microenvironment and survival time. It is a comprehensive resource for systematically researching immune infiltrates across diverse cancer types. As the cSCC database with clinic information was unavailable, head and neck squamous cell carcinoma (HNSCC) is often used as an alternative to cSCC in most studies [23, 24]. Thus, we used TCGA HNSCC data to explore the association between lymphatic-centered immune microenvironment and survival time. A total of 522 patients with HNSC and 44 regular patients were included in this study. The exploration module analyzed the clinical relevance of LYVE1 gene expression, CD8A^+^T cell infiltration, and CD8B^+^T cell infiltration in cSCC (Gene_Outcome). To analyze the differential expression between the tumor and adjacent normal tissue for VEGFC and YAP1 genes of interest across all TCGA tumors, we used the exploration module (Gene_DE). The tumor immune subsets' clinical relevance was analyzed by the resistant module (Outcome).

### Patient sample collection

Primary cSCCs and patient-matched standard adjacent samples for immunohistochemistry (IHC) staining were obtained from consecutive cSCC patients, all immunotherapy-naive. All diagnoses of cSCC were verified histologically by a board-certified dermatopathologist. The Institutional Review Board of Shanghai Skin Disease Hospital approved the study.

### Statistical analysis

Statistical analyses were performed using GraphPad Prism 9.5 (GraphPad Software). The one-way analysis of variance (ANVOA) was performed to compare the differences of epidermal thickness, dermal thickness, the collagen thickness, immune cells number or density, LV and BV number among different groups. The grade of collagen fiber disorganization among groups was analyzed with Kruskal–Wallis test. The T test was performed to compare the differences of LV and BV in human skin. The difference of VEGFC expression in kinds of cancers was determined by Wilcoxon test. The correlation was determined by the Spearman’s correlation test. Data were presented as means ± SEM, and differences were considered statistically significant when P ≤ 0,05. *: p-value < 0.05; **: p-value < 0.01; ***: p-value < 0.001; ****: p-value < 0.0001 indicated a significant difference among groups; ns, no significant difference among groups [30–32].

## Result

### The relevance between lymphatic-centered immune microenvironment and HNSCC patient cumulative survival time

**Fig.1:**
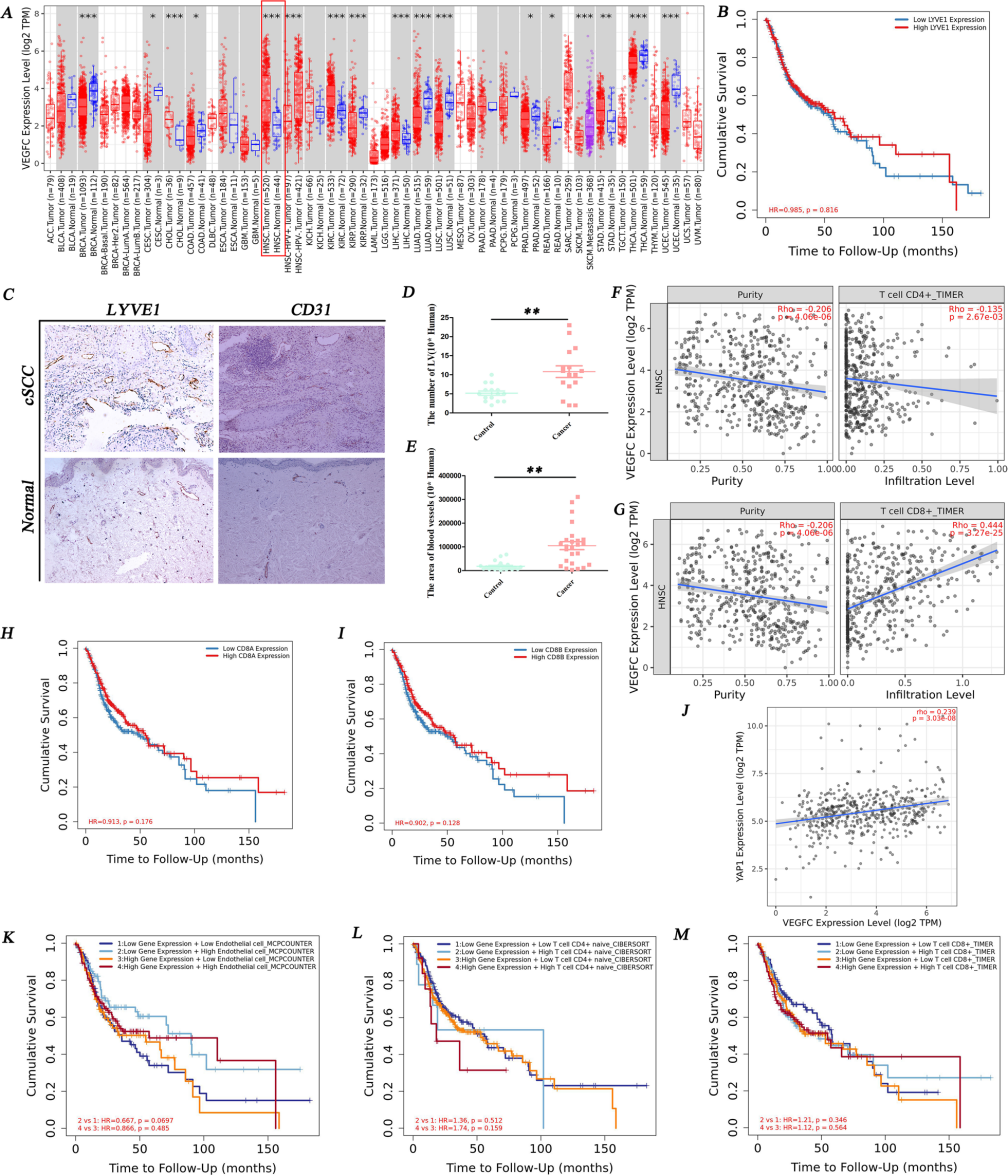
The relevance between lymphatic-centered immune microenvironments regulated by YAP1/VEGFC signal pathway and HNSCC patient cumulative survival time. **A** The heat map of VEGFC gene expression in HNSCC and healthy tissues. In HNSCC, the VEGFC gene showed an overexpression. P < 0.05; **B** The survival time relevance of LYVE1 gene expression analysis in cSCC was used by the exploration module (Gene_Outcome); **C** Image of LVs (LYVE1 with brown color, a particular marker of LVs) and BVs (CD31 with brown color, a unique feature of BVs) of human skin immunochemistry staining in the dermis. Magnification of microscopic image: 100 × . Statistics of the density of human LVs **D** and BVs **E** per area in immunochemistry staining. **F** The CD4^+^T cell and **G** CD8^+^T cell relevance of VEGFC gene expression in cSCC. The CD8^+^T cell relevance of VEGFC has a positive correlation. The CD4^+^T cell relevance of VEGFC has a negative correlation. P < 0.05; **H** The cumulative survival time relevance of CD8A^+^T cells and I CD8B^+^T cell infiltration in cSCC; **J** The association of VEGFC and YAP1 gene expression in HNSCC. They have a positive correlation. P < 0.05; When the YAP1 gene, endothelial cell **K**, immune cells **L** and **M** were in different expression or infiltration levels, respectively, the cumulative survival time of HNSCC patients differed. *, p < 0.05 and **, p < 0.01 indicated a significant difference between groups; ns, no significant difference among groups

Our study showed that the expression of VEGFC in HNSCC was significantly higher than that in normal tissues, meaning LVs might proliferate in tumors (Fig. 1A). Figure 1D confirmed that in human cSCC, the number of LVs was more than in healthy skin. Besides, BVs density also increased (Fig. 1E). The cumulative survival time of HNSCC patients with overexpressed LYVE1 gene is longer than those with low expression (Fig. 1B). The overexpression of the VEGFC gene was negatively correlated with the infiltration of CD4^+^T cells (Fig. 1F) and positively correlated with CD8^+^T cells (Fig. 1G), both of which were statistically significant. The high infiltration of CD8A^+^T cells and CD8B^+^T cells in HNSCC were conducive to prolonging the survival time (Fig. 1H, I). VEGFC acts as the upstream of YAP1 in amplification to increase lymphatic density in some malignant tumors [14]. However, no research has shown the relationship between YAP1 and VEGFC in cSCC. Our study showed that the expression of VEGFC was positively correlated with YAP1 in HNSCC (Fig. 1J), which was statistically significant, indicating that YAP1/VEGFC signal pathway might promote lymphangiogenesis in cSCC. When YAP1 gene overexpression and high infiltration of endothelial cells took place simultaneously might prolong the survival time of HNSCC patients (Fig. 1K). And high infiltration of CD8^+^T cells prolonged the survival time as well (Fig. 1M). However, the YAP1 gene overexpression and high infiltration of CD4^+^T cell might be unfavorable for the cumulative survival time (Fig. 1L). These preliminarily showed the critical role of lymphatic-associated immune environment in HNSCC via regulating the YAP1/VEGFC signal pathway, providing a direction for the timely intervention of lymphatics and related immune microenvironment to prolong survival time.

### UVR-induced alterations of skin appearance from healthy skin to photoaging skin and cSCC

**Fig. 2:**
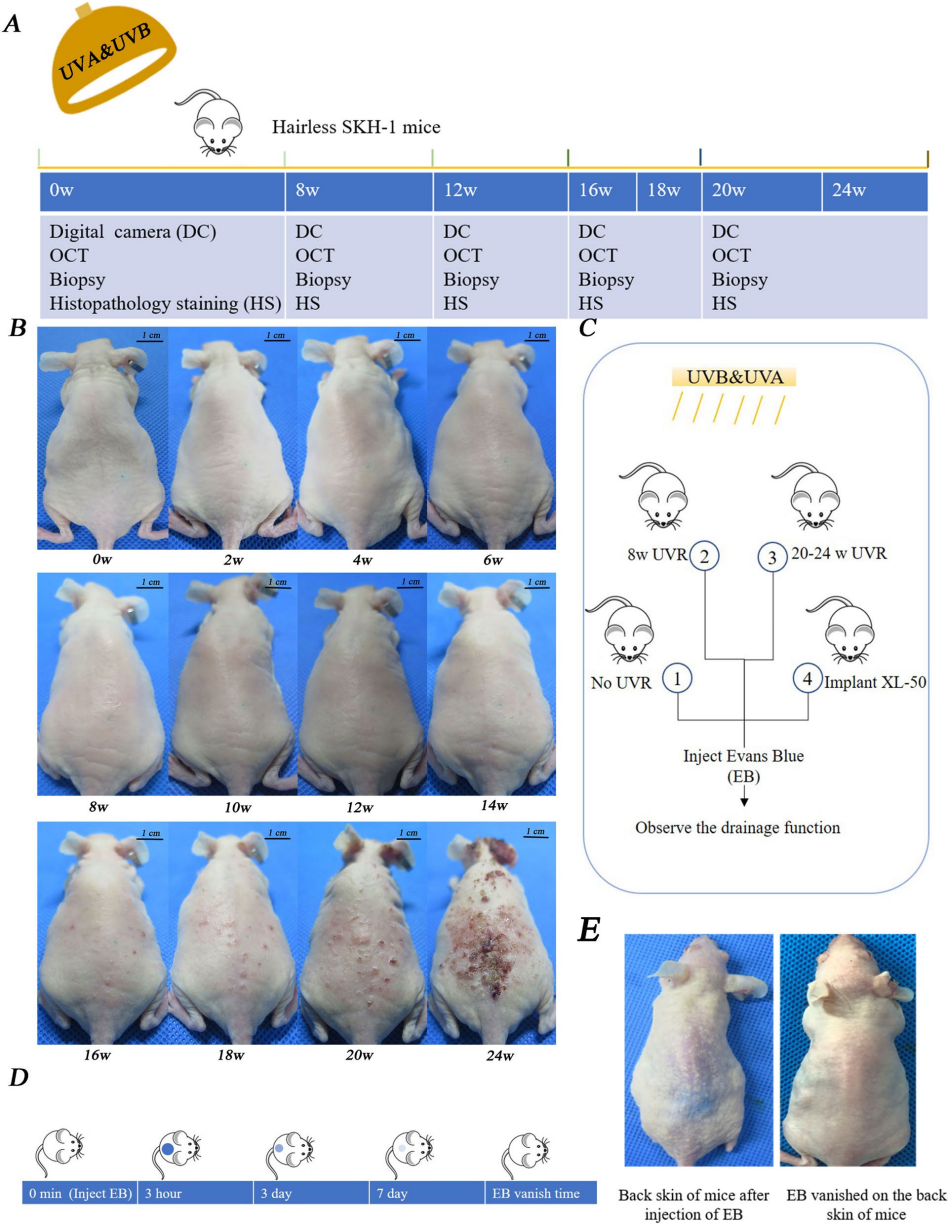
UVR-induced changes in skin appearance. Digital photographs of mice exposed to UV. **A** Diagram of experiment design of UVR cSCC mice. **B** Establishment of UV-induced cSCC mice model. By the eighth week of UVR, the skin showed typical photoaging manifestation. From the 12th to 14th week of UVR, needle-tip-size papules appeared. After 16 weeks of UVR, the number and size of papules increased. Exposure to UV for 20 weeks, cSCC lesions appeared. **C, D** and **E** Diagram of experiment design of Evans Blue assay. W, weeks; min, minute; h, hour; d, day

The solar simulator was used to establish the cSCC mouse model. Twenty SHK-1 Hairless mice were randomly divided into five groups (Fig. 2A). Besides, another 16 mice were divided into four groups to evaluate the drainage function of skin (Fig. 2C). They were injected EB and assessed at different times (Fig. 2D). After eight weeks of UVR, the skin of SKH-1 mice showed coarse wrinkles, pigmentation, and scales, which were the typical photoaging appearance. Then needle-tip-size papules gradually appeared. The skin became dry and lost its elasticity. From the 16th to 18th week, diffuse papules were observed, which gradually increased in number and size (Fig. 2B). According to the previous article published by Wang et al., this stage is precancerous, which is in line with the performance of AK [28]. After 20 weeks of UVR, cSCC lesions occurred [28, 33], shown in our study as well.

### The LV density decreased at the first turning point (UV-8w) and increased rapidly at the second turning point (UV-16-18w)

**Fig.3:**
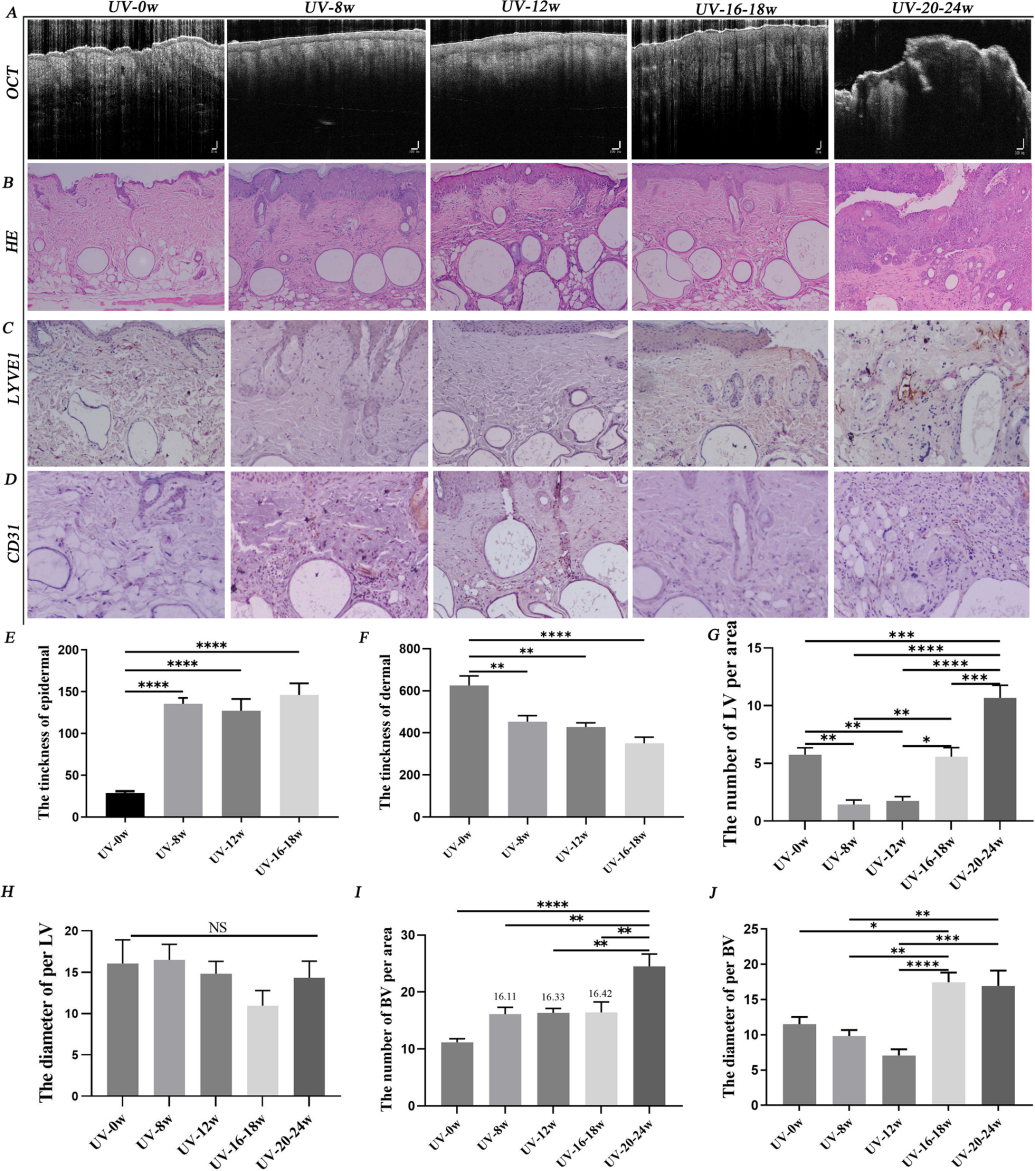
The dynamic changes of lymphatic density during the formation of cSCC. **A** OCT images of mice. **B** Histopathological results of mice. HE staining showed the thickness of the epidermis increased, and the dermis became thin after chronic UVR. **C **Image of LVs and **D** BVs immunochemistry staining in the dermis. **E** and **F** showed that the statistics of the epidermis and dermis thickness. Statistics of LVs **G** and BVs **I** density per area in immunochemistry staining. After eight weeks of UVR, the density of LV was at the lowest point. When AK lesions appeared, LV slightly out-numbered healthy skin. The lymphatic density in the dermis increased sharply when normal skin progressed into cSCC. The density of BVs increased rapidly when normal tissue progressed into photoaging, and AK lesions moved into cSCC. In immunochemistry staining, statistics of LVs **H** and BVs **J** diameter per area. Magnification of microscopic image: 100 × . *, p < 0.05 and **, p < 0.01 indicated a significant difference between groups; ns, no significant difference among groups

OCT in vivo imaging showed the epidermis became significantly thick from the 8th to 18th week of UVR (Fig. 3A). After 8th week of UVR, the epidermal thickness increased significantly. In the AK lesion, the dermal–epidermal junction (the horizontal low-density line) had partly disappeared, and the irregular wave-like shape of the dermal–epidermal junction was pronounced. In cSCC, the dermal–epidermal junction was no longer visible in some lesions (Fig. 3A). Histopathology confirmed this result, and the dermis gradually became thin (Fig. 3B, E, F). LVs and BVs work together to maintain interstitial fluid balance and immune homeostasis. LVs play an essential role in controlling UVB-induced edema formation and inflammation [34]. However, we did not know how the density of LVs and BVs altered during the formation of cSCC. The immunohistochemistry results showed that the number of LVs in photoaging skin (UV-8w) was the least. After a month of UVR, it gradually increased but was still lower than in healthy mice. When photoaging skin progressed to AK lesion (UV-16-18^th^w), the number of LVs increased and slightly more than that in healthy skin. When the cSCC lesions appeared, lymphatic density in sub-cancerous tissue rose sharply (Fig. 3C, G). The diameter of LVs was not statistically significant (Fig. 3H). According to our immunohistochemistry result, BVs density increased rapidly when healthy skin moved into photoaging (UV-8thw), and AK (UV-16-18thw) progressed into cSCC. It was almost unchanged from the photoaging to the precancerous stage. The diameter of the BVs became wider when they entered the precancerous state (Fig. 3I, J).

### The lymphatic drainage function declined at the first turning point (UV-8w) and restored when it progressed into cSCC

**Fig.4:**
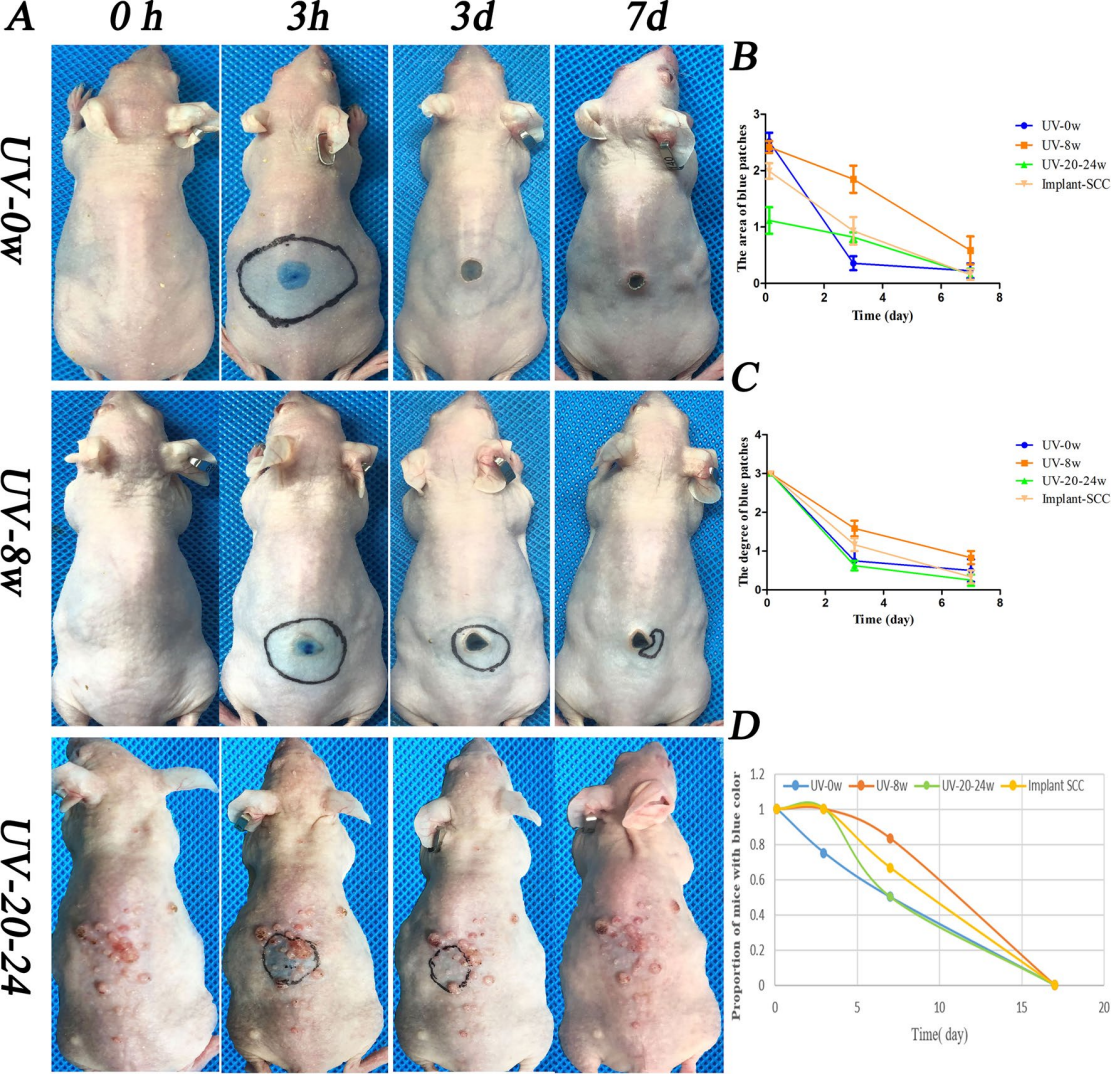
Chronic UVR-induced lymphatic drainage function declined in photoaging skin and restored in cSCC skin. UVR-induced drainage function of LVs assessed by Evans Blue assay in vivo. **A** Image of Evans Blue in the skin. **B** Statistics of the area of Evans Blue. Mice in the UV-0w group spent the shortest time drainage Evans Blue, UV-20-24w the second, implant SCC the third, and UV-8w the last. **C** Statistics of the Evans Blue grade in different time. The blue color grades in the UV-20-24w group appeared as the lightest color, UV-0w the second, and UV-8w the darkest shade. **D** The number of mice with some residues was shown in blue color. The horizontal axis represents time (day)

To evaluate the drainage function of LVs in cSCC induced by UVR, we performed the Evans Blue experiment in vivo. According to the area of blue patch, the blue grade of the patch, and the number of mice with residual blue patches, the clearance rate of LVs was observed quantitatively. The clearance rate of UV-0w group mice was superior to other groups. UV-20-24w (cSCC mice) group and implant cSCC mice took second and third place, respectively. The drainage function of photoaging mice was the worst among these group mice (Fig. 4B, D). It probably indicated that during the formation of cSCC induced by UVR, the number and function of LVs gradually adapted. In other words, to effectively cope with the occurrence and development of cSCC, LVs increased, and the total drainage function improved or maintained the original level as much as possible. The drainage function of cSCC skin was still lower than in healthy mice when the number was highly exceeded. Thus, the average LVs’ function might decline significantly, and the non-functional LVs increased.

### The dynamic changes of immune cell infiltration along with lymphatic function and quantity changes during the formation of cSCC induced by UVR

**Fig.5:**
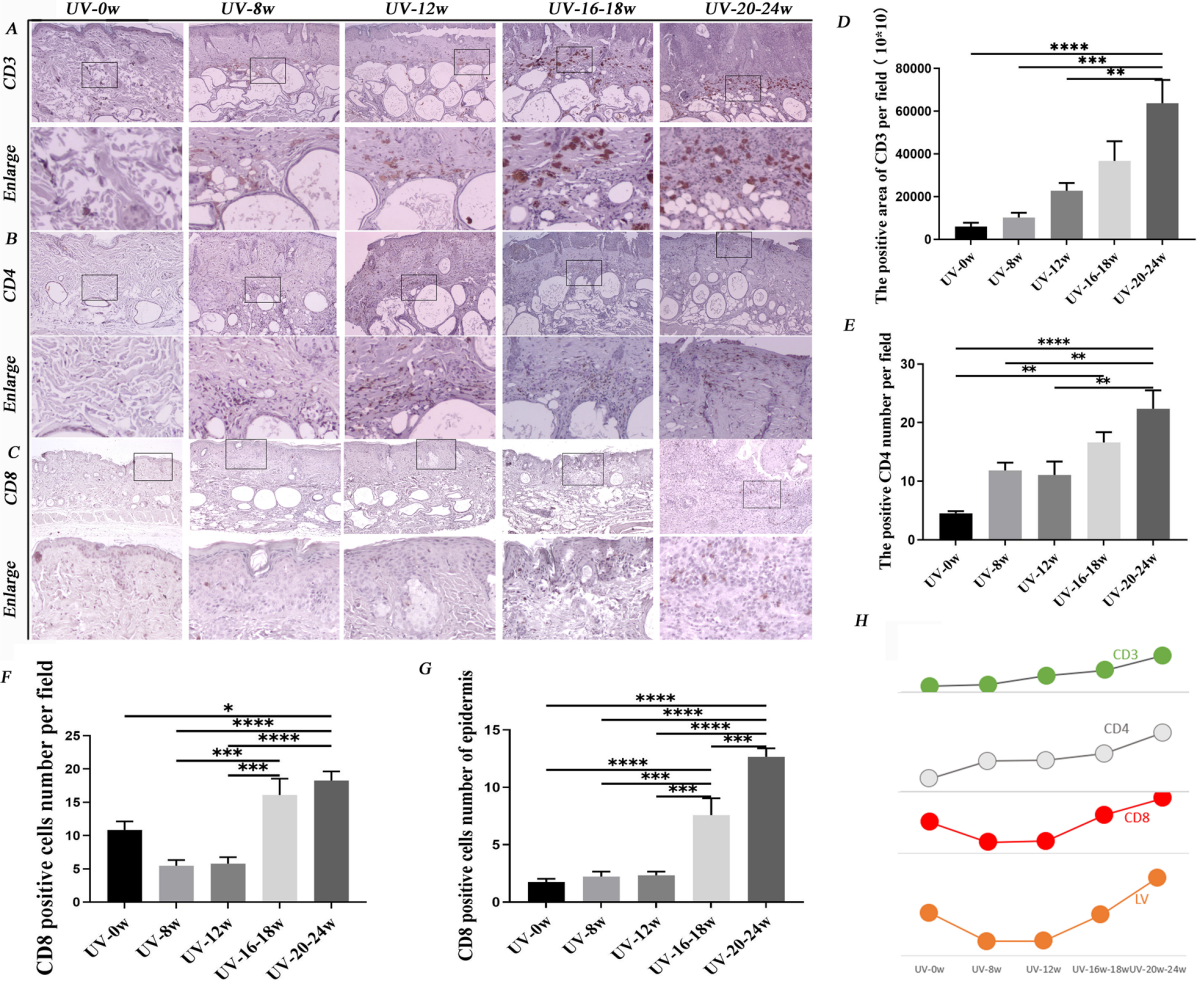
UVR-induced immune cell infiltration during the formation of cSCC. **A** Immunochemical image of CD3; **B **Immunochemical image of CD4; **C** Immunochemical image of CD8; **D** Statistics of CD3 positive cells. After 12 weeks of UVR, the number of T cell increased and again grew significantly when the AK lesion developed into cSCC; **E** Statistics of CD4 positive cells. The CD4^+^T cells increased rapidly in photoaging skin and almost kept the quantity until AK occurred. When AK developed into cSCC, it rose significantly; **F** Statistics of CD8 positive cells in mice skin. The infiltration of CD8^+^T cells reduced in photoaging skin. When AK occurred, CD8^+^T cells sharply increased, especially in the epidermis; **G** Statistics of CD8 positive cells in epidermis; **H** The trend lines of CD8^+^T cell, CD4^+^T cell, CD3^+^T cell, and LVs value. Magnification of microscopic image: 100 × . *, p < 0.05 and **, p < 0.01 indicated a significant difference between groups; ns, no significant difference among groups

In chronically UVR skin, there are more immune cells than in photo-protected skin [8]. LVs and immune cells in human metastatic cutaneous cancer have strong correlations [8]. Our experiment showed that the proliferation of LVs in HNSCC might be related to CD4^+^T cell and CD8^+^T cell infiltration (Fig. 1F, G). Nevertheless, we did not know what the immune system, especially immune cells, with LVs as the center, experienced during the formation of cSCC induced by UVR. IHC experiments were conducted by labeling with CD3^+^, CD4^+^, and CD8^+^T cells to explore these questions. The total number of T cells (CD3 positive) increased slowly with accumulative UVR (Fig. 5D). CD4^+^T cells increased sharply in photoaging skin and maintained the same quantity until precancerous lesions appeared. When AK developed into cSCC, it proliferated (Fig. 5B, E). CD8^+^T cell reduced rapidly after eight of UVR (Fig. 5C, F). When AK occurred, it rapidly increased, especially in the epidermis (Fig. 5C, F, G). The alteration trend of CD8^+^T cells was similar to LVs density, while the CD3^+^T cell and CD4^+^T cell differed (Fig. 5H).

### UVR-induced dynamic changes of LVs involved in photoaging progress to cSCC via regulation YAP1/Piezo1

**Fig.6:**
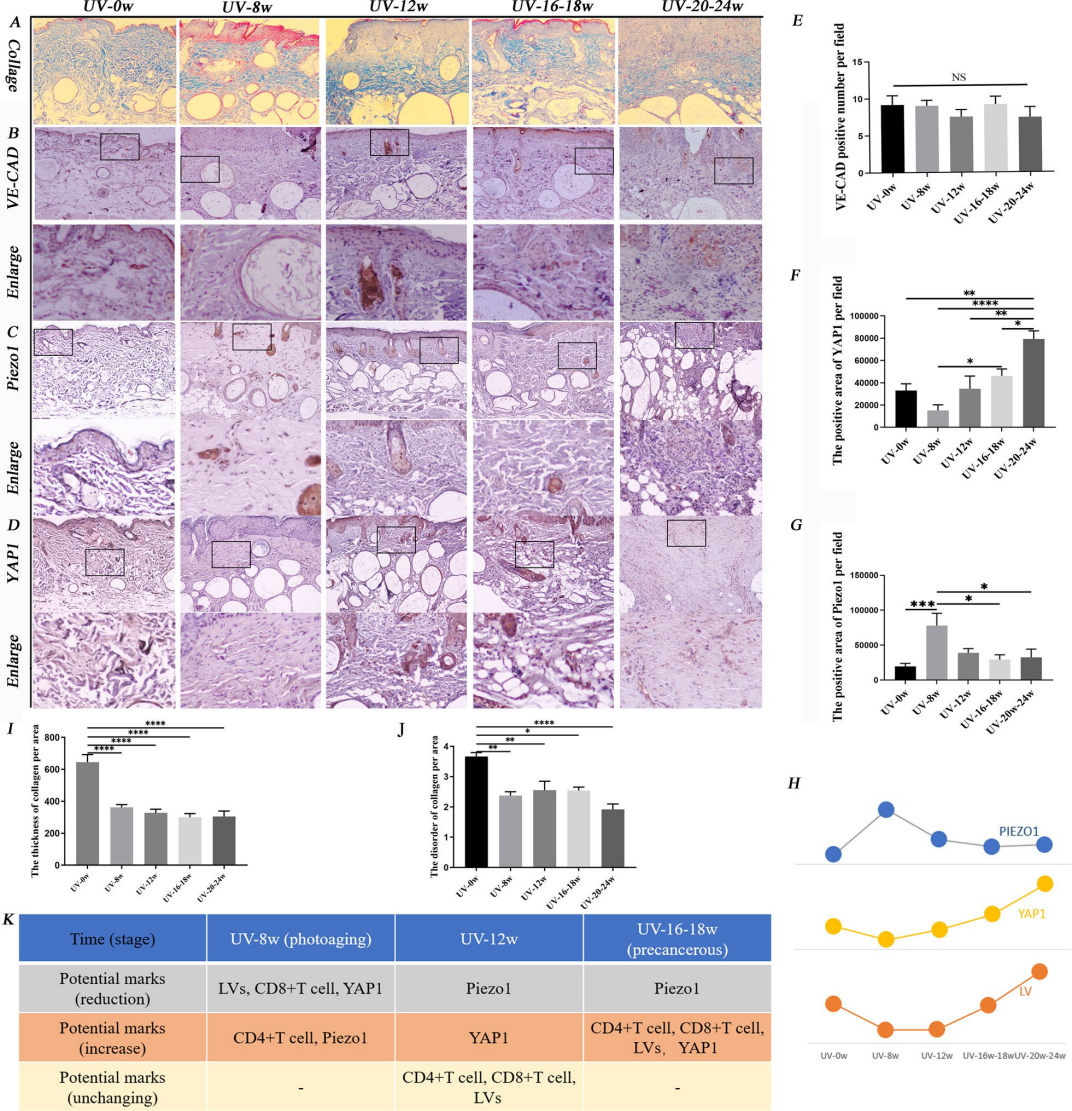
UVR-induced dynamic alterations of lymphatic structure and function regulated by YAP1/Piezo1. **A** Image of Masson staining. Magnification of microscopic image: 100 × . **B** Image of VE-CAD (VE-CAD with brown color), **C** Piezo1 (Piezo1 with brown color), and **D** YAP1 (YAP1 with brown color) immunochemistry staining in the dermis. Statistics of the area of VE-CAD **E**, YAP1 **F**, and Piezo1 **G** per area in immunochemistry staining. The expression of YAP1 after eight weeks of UVR was the least. When normal tissue progressed to AK, it exceeded normal skin. When AK moved into cSCC, the positive area increased rapidly. Piezo1 represented the opposite trend compared with YAP1. **H** The trend lines of YAP1, LVs, and Piezo1 value. I Statistics of collagen thickness. **J** Statistics of the grade of collagen disorganization. Magnification of microscopic image: 100 × . **K** The potential lymphatic-centered immune microenvironment markers in different skin stages induced by UVR. *, p < 0.05 and **, p < 0.01 indicated a significant difference between groups; ns, no significant difference among groups

**Fig.7:**
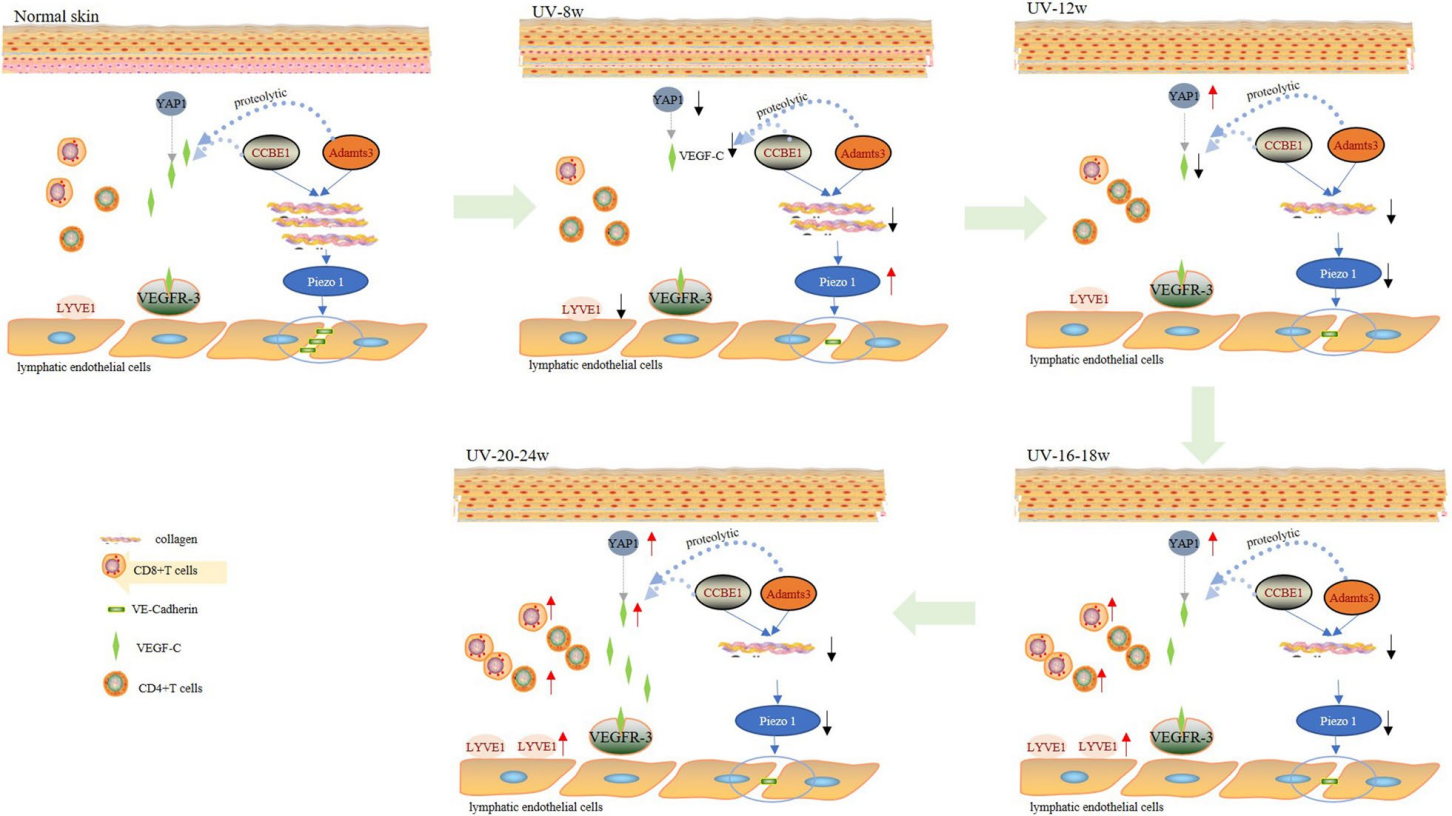
Diagram that represents the mechanisms of lymphatic-centered immune microenvironment dynamic changes during the formation of cSCC. By adjusting YAP1/VEGFC and Piezo1, the number and function of LV were adaptable under continuous UVR. Two key periods were UVR-induced eight weeks (photoaging) and 16–18 weeks (precancerous). After 8 weeks UVR, Piezo1 expression surged. However, because to a drop in YAP1 expression, the density of LVs in UV-8w dramatically decreased. Meanwhile, the collagen arrangement loosened over time and the overall amount reduced. As a result, the lymphatic drainage function declined, which Evans Blue assay validated. Lymphatic dysfunction initiate may active CD4^+^T cells to impair lymphatic function. Additionally, UVR activated suppressive CD4^+^T cells and stopped the infiltration of antigen-specific CD8^+^T cells, ultimately suppressing cellular immunity (photoimmunosuppression). After 12 weeks of UVR, YAP1 marginally increased. YAP1 expression spiked when AK lesions occurred, increasing LV density as a result. The pace of Piezo1 reduction slowed down and began to settle into a platform. Although Piezo1 was greatly reduced in the cSCC stage, the drainage and pump functions were better than those of photoaging skin because YAP1 increased, which caused the lymphatic density to improve. In the UV-16-18w and UV-20-24w groups, the infiltration of CD8^+^T cells and CD4^+^T cells significantly increased

Collagen is a vital tissue that maintains the pumping ability of LVs and BVs [35]. The collagen arrangement gradually loosened and the total amount decreased. In UV-8w group, some collagen began to curl and thicken into clumps. In UV-12w group, the collagen bundles gradually became thinner (Fig. 6A, I, J). The negative changes of collagen caused by UVR might induce the dysfunction of LVs. Our study showed that VE-CAD in healthy skin was higher than in photoaging and cSCC (Fig. 6B). However, it was not statistically significant (Fig. 6E). The trend of YAP1 protein expression was almost consistent with LVs density during the formation of cSCC (Fig. 6D, F). After eight weeks of UVR, the expression of YAP1 decreased and was at its lowest level during the formation of cSCC. After one month, it started to go up. When AK lesions appeared, it sharply rose (Fig. 6F). This indicated that YAP1 might be one primary driver which regulated lymphatic density during the formation of cSCC induced by UVR. Piezo1 controls lymphatic valve development and maintenance [17, 18]. The expression of Piezo1 represented the opposite trend compared with YAP1 and LVs. In photoaging skin, it overexpressed, then reduced. When the skin developed into AK lesions and progressed into cSCC, the expression of Piezo1 reduced slowly (Fig. 6C, G). In conclusion, with the accumulation of UVR, lymphatic density, VE-CAD, and Piezo1 experienced dynamic changes (Fig. 7). LVs, CD8^+^T cells, and YAP1 were most inhibited in photoaging skin. CD4^+^T cells and Piezo1 expression increased. Then YAP1 slightly increased, but LV and CD8^+^T cells were still in the inhibition state in UV-12w group. LV, BV, CD4^+^T cell, CD8^+^T cell, and YAP1 significantly increased when cSCC lesion appeared (Figs. 6K and 7).

## Discussion

There was no research on what constant changes of LV and the lymphatic-centered immune microenvironment experienced while healthy skin developed into photoaging, eventually to cSCC. This study showed that the lymphatic-centered immune microenvironment was adaptive to continuous UVR via regulating YAP1/VEGFC and Piezo1. Besides, photoaging and precancerous were two turning points during the formation of cSCC induced by UVR. YAP1, LVs, and immune cells can be used to identify the skin lesion stage and take action to prevent cSCC in time. This study offered a specific clinical significance for the early diagnosis of cSCC and prognosis judgment. Besides, it provided a theoretical basis for studying the pathogenesis of cSCC.

Skin provides physical, chemical, and microbiome barriers against harmful factors from the environment [32]. Chronic UVR leads to the accumulation of oxidants, SASP, and inflammatory factors, which promotes the process of photoaging, whole-body aging, and progresses into cSCC eventually (Fig. 2B). Previous studies showed that low ROS level was essential for lymphangiogenesis. On the contrary, oxidative stress due to enhanced ROS generation or reduced levels of antioxidants suppressed lymphangiogenesis via promoting LECs apoptosis and death [36, 37]. However, LVs density increased in human and mice cSCC (Fig. 1C). In HNSCC, VEGFC gene expression was significantly higher than in normal tissue (Fig. 1A). According to this study, the number and function of LV were adaptive under continuous UVR via regulating YAP1/VEGFC and Piezo1. First, the function of LVs in UVR induced cSCC was significantly better than the SCC cellular implanted cSCC (Fig. 4). This indicated the relevant mechanisms regulating the function and the quantity of LVs might have undergone adaptive changes during long-term chronic UVR. YAP1 is the downstream of VEGFC. The VEGFC and YAP1 gene expression were also positively correlated in HNSCC tissues (Fig. 1J). Meanwhile, our animal experiments showed that the increase of YAP1 expression preceded the expansion of lymphatic density, and there was a positive correlation between them, which indicated that YAP1/VEGFC signaling pathway was a critical regulatory signal to promote lymphangiogenesis. The proteins related to the lymphatic function, such as Piezo1, VE-CAD, and collagen, might also undergo continuous dynamic changes. After eight weeks of UVR, Piezo1 expression peaked (Fig. 6C, G). Although Piezo1 is overexpressed, the LVs density and VE-CAD expression were the lowest in the UV-8w group. Besides, the collagen arrangement gradually loosened and the total amount decreased. Thus, the lymphatic drainage function declined in the UV-8w group, which confirmed by Evans Blue assay (Fig. 4). Upon progressing to AK (UV-16-18w) and cSCC, LVs density and YAP1 expression increased rapidly. The speed of Piezo1 reduction slowed down and tended to in a platform (Fig. 6G). In the cSCC stage, though Piezo1 significantly reduced (Fig. 6G), the drainage function and pump function of cSCC were better than photoaging skin (Fig. 4), because of the lymphatic density increase (Fig. 3G). These might explain why more LVs were beneficial in prolonging the survival time of cSCC patients (Fig. 1B). We speculated that the balanced of Piezo1-YAP1/VEGFC in preserving the lymphatic density and drainage function might be beneficial to prevent cancer progression. When the balance breaks, cSCC may further develop. Previous studies have shown that the Hippo pathway typically regulated activity by limiting the entry of YAP and TAZ (Transcriptional coactivator with PDZ-binding motif) into the nucleus, and the deregulation of the hippo pathway has been reported at a high frequency in a broad range of different human carcinomas, including lung, colorectal, ovarian, liver and prostate cancers [15]. Besides, YAP overexpression promoted oral squamous cell carcinoma cell growth [38]. However, there is limited research related the impact of YAP1/VEGFC signaling pathway on the formation of cSCC. According to our study, after acute UVR, low expression of YAP1/VEGFC maximized the reduction of inflammatory edema, while long-term UVR, YAP1/VEGFC overexpression promoted lymphangiogenesis and played an immune response role. During the formation of cSCC, on the one hand, YAP1 combined with VEGFC to promote lymphatic development and generate anti-cancer cells, and on the other hand, it may combine with TAZ to promote the proliferation of cSCC cells. YAP/TAZ and YAP1/VEGFC may exists a balance controlling cSCC progress, this still needs further exploration.

In the process of cSCC formation, the skin microenvironment has taken place with significant changes, such as collagen (Fig. 6A). The total amount of collagen bundles decreased and arranged in disorder. Before the 20–24th week of UVR, some collagen began to curl and thicken into clumps. The study confirmed that skin stiffness was conducive to tumor growth and metastasis [39]. The thicker bundle of collagen traps immune cells, which induces cytokine accumulation and weakly responds to external stimulation. In another way, collagen is a vital tissue that maintains the pumping ability of LVs and BVs [35]. The negative change in collagen caused by UVR induced the dysfunction of LVs [35, 40], which might lead to an ineffective immune response. UVR has a significant impact on immune regulation, such as photoimmunosuppression [41]. However, there is currently no experiment to show how immune cells, especially T cells, change during the formation of cSCC induced by long-term UVR. In our study, T cells experienced adaptive change. The total number of T cells increased slowly before cSCC appeared (Fig. 5D). CD4^+^T cells rapidly reproduced after the eighth week of UVR (Fig. 5). Krasteva et al. [42] found that CD4^+^T cells were one of the target cells of UVB-induced immune suppression in the skin or the body. UVR activated suppressive CD4^+^T cells and prevented the proliferation of antigen-specific CD8^+^T cells under its mediation, ultimately inhibiting cellular immunity. These explained why CD4^+^T cells proliferated and CD8^+^T cells shrank in number or were unchanged after eight weeks of UVR (Fig. 5E, F). Besides, lymphatic dysfunction initiate may active CD4^+^T cells to impair lymphatic function [43]. A study showed that upon lymphatic injury, CD4^+^T cells get activated into a mixed Th1 and Th2 phenotype by dendritic cells in the regional lymph nodes and then migrate to the injury site to initiate lymphedema pharmacological inhibition of T cell release from the lymph nodes. This resulted in reduced lymphedema, suggesting that CD4^+^T cells impair lymphatic function after lymphatic injury [44]. Another study showed that T cells negatively regulated lymphatic function and lymphangiogenesis was through IFN-γ secretion, leading to the suppression of lymphatic-specific genes in LECs and consequently causing marked reduction in lymph node lymphangiogenesis [45]. This might be one reason that the lymphatic density was the lowest and its function worst in the initial UVR (8th week) (Fig. 4B, C, D). Meanwhile, the time of immunosuppression induced by UVR might last 12 to 15 weeks (from 0 weeks to 12 or 15 weeks). A previous study showed that photoimmunosuppression intended to attenuate the UVB inflammation initially. Still, it might serve as the malignant cell escape the immune surveillance leading to the occurrence of cSCC. While UV stimulation continues and precancerous lesions (UV-16-18w, AK lesions) appear, killer and regulatory lymphocytes proliferate, especially in the epidermis (CD8^+^T cells) (Fig. 5F, G). This time the dominant role might be active CD4^+^T cells rather than suppressive ones. CD8^+^T cells and CD4^+^T cells worked together to maintain the extracellular environment’s stability and inhibit tumors’ occurrence and development. Timer2.0 indicated a negative correlation between VEGFC gene expression and CD4^+^T cells in HNSCC tissue, with a low correlation. In animal experiments, AK lesions progressed to cSCC and CD4^+^T cells more infiltrated. With the continuous stimulation of UV, in order to cope with the occurrence of other malignant diseases such as cSCC, LECs need to present antigens to CD4^+^T cells and regulate the immune response to kill cancer cells [46]. This may indicate that other signaling pathways are activated during the early stages of cSCC formation, leading to a significant increase in CD4^+^T cell infiltration and exerting anti-tumor effects. Another possibility is that with the continuous progress of cSCC, the number of CD4^+^T cell infiltration may decrease or the function change to inhibitory regulation, and the microenvironment will gradually enter the state of immunosuppression or immune tolerance. TIMER2.0 showed that VEGFC gene expression and CD8^+^T cell infiltration positively correlated in HNSCC (Fig. 1G). The high infiltration of CD8A^+^T and CD8B^+^T cells in HNSCC prolonged the survival time (Fig. 1H, I). At the same time, high expression of YAP1 with increased infiltration of CD8^+^T cells and endothelial cells in HNSCC improved the survival rate of patients (Fig. 1L, M). Our animal study showed that the trend of CD8^+^T cells was almost consistent with the LVs and YAP1 (Figs. 5H, 6H). This confirmed that LV had a strong relationship with immune cell infiltration in cSCC and indicated that LV and immune cell infiltration are consistent throughout the formation of cSCC induced by UVR, especially in skin aging and precancerous lesions.

BVs play essential roles in the skin extracellular matrix, providing energy and oxygen, which depend on vascular functionality and metabolic demand [47–50]. In photoaged skin, the number of BV in the upper dermis reduced, with the dilatation of remaining vessels [40]. These might be caused by severe photodamage. However, our result seemed different; when healthy skin developed into photoaging, BV increased significantly, and we did not find apparent dilatation of remaining vessels. These phenomena might result from small samples and sample heterogeneity. When the normal skin developed into AK, the number of BV did not increase much. At the same time, the diameter dilatated a lot, indicating that the pronounced BVs might act as a precursor to progression into cSCC (Fig. 3D, I, J). When cSCC lesions appeared, BVs in tumors increased and showed prominent dilatation (Fig. 3I, J). BVs in tumor tissues were far from the average [51–53], such as aberrant and leaky, loose endothelial junctions, a discontinuous endothelial lining, and a defective basement membrane. These features are ultimately all signs of poor vessel maturation and functionality, with the consequence that a tumor constantly remains hypoxic, which leads to a negative feedback loop whereby proangiogenic signals never stop. These explained why BV increased when cSCC appeared. A large number of abnormal BVs enable a more remarkable extent, cancer cells to sneak into the circulation and metastasize to distant organs [43, 54, 55]. If this malignant negative feedback can be effectively interrupted when AK appears, it may be beneficial to prevent cSCC and metastasis.

As long-term UVR is the most important risk factor for the cSCC [56], and UVR induced cSCC modeling technology is mature and highly recognized, our study selects UVR modeling to evaluate the dynamic changes of microenvironment during the formation of cSCC. However, in addition to UVR, there are also some risk factors that have not been considered. Organ transplantation (ORT) with long-term use of immunosuppressants recipients [57], infected with HPV, HIV and other viruses, exposed to chemical carcinogen such as arsenic, suffering from xeroderma pigmentosum, eye skin albinism and other genetic diseases will increase the risk of suffering from cSCC [58]. Part of these risks are due to immune suppression leading to an increase in the prevalence of cSCC, such as ORT. cSCC patients with immune suppression are more prone to transit metastases [59]. The inflammation formed around the foci of the SCC in OTRs is less intense and the number of CD3^+^ T and monocytes was smaller, which may be responsible for the tumor aggressiveness [60, 61]. The cutaneous β HPVs are hypothesized to contribute to pathogenesis of cutaneous SCC [62]. However, recently, a study showed that HPV infection might relieve the effects on them of sun exposure [63]. It might enhance the effect of CD8^+^ T cells to reach, but there is no direct evidence to prove. Xeroderma pigmentosum (XP) is a rare autosomic recessive disorder of DNA repair pathway that confers photosensitivity along with an increased incidence of UV-induced cSCC [64]. These factors might influence the immune microenvironment, but there is little research related to it. Future research could incorporate experimental designs, such as gene knockout or pathway inhibition, to confirm the role of identified factors.

This study mainly observed the dynamic histopathological alterations in the lymphatic-centered immune microenvironment during the formation of cSCC induced by UVR. The lymphatic-centered immune microenvironment is adaptive under UVR via regulating YAP1/VEGFC and Piezo1. UVR yielded 8 and 16–18 weeks in this process, two key turning points. UV-8w represented photoaging skin, the lymphatic function and some immune cells were in a suppressive state. After eight weeks of UVR, YAP1 depression inhibited cutaneous lymphatic angiogenesis. Inhibitory CD4^+^T cell proliferation and LVs reduction might contribute to CD8^+^T cell decrease and distribution. When skin moved into the precancerous state, YAP1, CD8^+^T, CD4^+^T, CD3^+^T, and LVs surged, and Piezo1 gradually decreased (Fig. 7). When YAP1 slightly increased and Piezo1 reduced, without intervention, the photoaging skin might progress into precancerous lesions, even cSCC. More functional LVs and more CD8^+^T cell infiltration might prolong the cumulative survival time of cSCC patients.

## Data Availability

Please contact the corresponding author (e-mail address: wangpeiru@tongji.edu.cn).

## Abbreviations

UVR: Ultraviolet irradiation
OCT: Optical coherence tomography
cSCC: Cutaneous squamous cell carcinoma
HNSCC: Head and neck squamous cell carcinoma
LV: Lymphatic vessel
AK: Actinic keratosis
LECs: Lymphatic endothelial cells
VEGFR-3: Vascular endothelial growth factor receptor-3
LYVE1: Lymphatic vessel endothelial hyaluronan receptor 1
VEGF-C: Vascular endothelial growth factor C
Piezo1: Piezo-type mechanosensitive ion channel component 1
YAP1: Yes-associated protein 1
ROS: Reactive oxygen species
ORT: Organ transplantation
